# Early bilingualism as a protective factor against acute post-stroke aphasia

**DOI:** 10.1007/s00415-026-13820-2

**Published:** 2026-04-27

**Authors:** Marcello Naccarato, Federica Palacino, Edoardo Ricci, Ilario Scali, Magda Quagliotto, Michele Malesani, Gianpiero Farina, Emanuele Vincis, Paola Potente, Martina Maset, Paola Caruso, Giovanni Furlanis, Paolo Manganotti

**Affiliations:** Neurology and Stroke Unit, Department of Medicine, Surgery and Health Sciences, University Hospital and Health Services of Trieste, University of Trieste, Friuli-Venezia Giulia, Strada di Fiume, 447, 34149 Trieste, Italy

**Keywords:** Aphasia, Aphasia recovery, Bilingualism, Post-stroke recovery

## Abstract

**Background and aims:**

Aphasia is among the most debilitating post-stroke deficits. Previous studies have suggested that bilingualism, the ability to use more than one language, may confer advantages in language recovery compared with monolingualism in patients with chronic stroke. Our aim was to determine whether early pre-scholar simultaneous bilingualism acts as a protective factor by examining language recovery within the first seven days after acute ischaemic stroke.

**Methods:**

We retrospectively analyzed clinical and neuroimaging data from Italian monolingual and early bilingual (Slovenian- and Croatian-Italian) patients with anterior-circulation ischaemic stroke and aphasia who were consecutively admitted between January 2018 and April 2020. The two cohorts were compared, and a multivariate logistic regression model was used to identify variables associated with language improvement at 7 days, defined as a ≥ 1-point reduction or complete recovery.

**Results:**

The two groups did not differ in demographic or clinical characteristics, type of acute treatment, or extent of the ischaemic lesion. Early bilingual patients exhibited significantly greater improvement in NIHSS language scores at day seven. In multivariate analysis, early bilingualism (p = 0.005) emerged as independent predictor of early language recovery, with consistent effect sizes across sensitivity analyses.

**Conclusions:**

Early bilingualism (eBL) is an independent and robust predictor of aphasia recovery within the first seven days after stroke.

**Supplementary Information:**

The online version contains supplementary material available at 10.1007/s00415-026-13820-2.

## Introduction

Aphasia occurs in approximately 30% of acute stroke patients [1] and represents one of the most disabling neurological sequelae, with major consequences for functional independence and quality of life [2–4]. People with aphasia present significant clinical heterogeneity with varying degrees of difficulty in the production and comprehension of spoken and written language; these deficits affect the clinical outcome and quality of life of patients with cerebrovascular injury [5–7]. In this context the profile of language impairment and the trajectory of aphasia recovery are complex and remain difficult to predict. The variability reflects the interaction of several determinants, including demographic characteristics, pre-stroke functional status, early hemodynamic changes, and lesion-specific features such as location, extent, and the degree of structural and functional disconnection. Despite this complexity, most studies on prognostic factors in aphasia have been conducted in monolingual stroke populations, even though bilingualism, defined as the routine use of two languages, is highly prevalent worldwide [8]. Whereas in monolingual individuals the clinical course of post-stroke aphasia is primarily driven by lesion topography and network disruption, bilingual individuals typically exhibit impairments across all spoken languages following acute stroke [7, 9].

Research on multilingual aphasia has proposed several theoretical frameworks to explain cross-linguistic differences in impairment. Early hypotheses suggested that either the first-acquired language (L1) or the most frequently used language would be preferentially preserved [10, 11]. However, while many studies have examined bilingual aphasia from the perspective of cognitive control and executive dysfunction [12, 13], evidence regarding the acute phase of aphasia in bilingual patients remains limited. Recent findings suggest that bilingualism may be associated with more favorable language recovery profiles than monolingualism in chronic post-stroke aphasia [9]. Moreover, neuroimaging studies indicate that early pre-scholar simultaneous bilingualism (eBL), in contrast to later sequential bilingualism, is associated with more integrated and efficient language and executive control networks [14, 15]. The distinctiveness of Trieste district is the historical presence of a large simultaneous “early” bilingual Slovenian-Italian and, to a lesser degree, Croatian-Italian minorities. The aim of this study was therefore to determine whether bilingualism represents a protective factor in acute stroke aphasia, by assessing language recovery based on improvement in the NIHSS language score at 7 days.

## Materials and methods

### Study population

We retrospectively analyzed clinical and imaging data of patients with acute ischemic stroke admitted to the Stroke Unit of the University Medical Hospital of Trieste (Italy) between January 2018 and April 2020. We included patients with a first-ever ischaemic stroke in the anterior circulation who presented with aphasia, as quantified by the NIHSS language sub-item [16]. Early bilinguals (eBL) were defined as individuals belonging to the recognized local linguistic minorities who acquired their L1 (typically Slovenian or Croatian in our district) and Italian (L2) simultaneously during early childhood, prior to school age.

Early bilingual patients belonged to the officially recognized Slovenian–Italian and Croatian–Italian linguistic minorities of the Trieste province. In this sociolinguistic context, both Italian and Slovenian or Croatian are used across formal, institutional, and healthcare settings, as well as within family and social environments. Both languages are acquired simultaneously in early childhood and maintained throughout the educational pathway. Language impairment and recovery were assessed using the NIHSS language sub-item, which was administered in Italian for all patients.

Exclusion criteria were: (1) posterior circulation stroke; (2) lacunar stroke (SVO); (3) hemorrhagic stroke; (4) stroke mimics and (5) monolingual non-Italian patients, as well as late (sequential) bilingual patients or with language combinations not including Italian as L1 or L2. All patients underwent non-contrast CT (NECT) at admission and a follow-up NECT scan 24–72 h after stroke onset. No age or sex restrictions were applied. Moreover, a comprehensive diagnostic workup, including assessment of vascular risk factors, neurological examination, laboratory tests, and instrumental investigations (electrocardiogram, carotid ultrasonography, transthoracic or transesophageal echocardiography, Holter-ECG monitoring, electroencephalography) to determine the stroke etiopathogenesis was performed. All types of treatment, including thrombolysis, thrombectomy, or medical treatment, were performed according to national guidelines.

Patients included in the study were therefore divided into two different groups: the group of eBL patients and the group of Italian monolinguals (ML) patients to identify predictors of early language improvement.

The research was conducted in line with the principles of the Declaration of Helsinki and was approved by the Local Ethics Committee CEUR (Comitato Etico Unico Regionale, FVG, Italy) with approval number 115/2018.

### Clinical data

Among the clinical data collected, we recorded: (1) demographic information (age, sex), spoken languages and education; (2) vascular risk factors, including atrial fibrillation (AF), diabetes mellitus (DM), arterial hypertension (AH), dyslipidemia and clinical status including pre-stroke independence and pre-stroke cognitive impairment; (3) stroke characteristics (National Institutes of Health Stroke Scale (NIHSS) [16] at admission and at discharge, ischemic stroke volume); (4) reperfusive treatment (intravenous thrombolysis (IVT), endovascular thrombectomy (EVT), IVT + EVT and time from stroke-onset to treatment); (4) hemorrhagic transformation (according to the SITS-MOST definition [17]; (5) clinical classification according to the Oxfordshire Community Stroke Project classification [18]; (6) outcomes: aphasia improvement defined as an at least 1-points decrease on 7-days-follow-up NIHSS language sub-item or full speech recovery [19], 90 days disability using mRS score [20], discharge destination and 90-days mortality.

### Neuroimaging assessment

Acute and 24/48-h follow-up CT brain scans were obtained according to normal clinical routine with a 256-slices CT scanner (Brilliance iCT; Philips Medical Systems, Best, Netherlands). Non-enhanced-CT were acquired with 120 kV, 400–450 mAs, at a slice thickness of 0.9 and reconstructed at 5 mm. Follow-up images were qualitatively assessed by trained neurologists (GF, PC) to confirm the diagnosis. All doubts were decided by consensus with a third neurologist (PM). Ischemic lesions were manually segmented (FP, ER) with MRIcroGl 1.2 and their volume was calculated in mL for each patient.

### Statistical analysis

We performed statistical analyses using SPSS Statistics 23 (IBM, Armonk/NY, USA). Kolmogorov–Smirnov test was used to evaluate the normal distribution of variables. Continuous variables with a normal distribution are presented as mean and standard deviations (SDs), those with a skewed distribution as median and interquartile ranges (IQRs) indicating the first and third quartiles, and categorical variables as counts and percentages (%). The two subgroups were divided into monolingual (ML) and early bilingual (eBL). Differences between groups were tested with Student’s t test for normally distributed continuous variables, Mann–Whitney U test for skewed variables, and Pearson’s Chi-square for categorical variables. Associations between continuous or ordinal variables were assessed using Spearman’s rank correlation coefficient (ρ), given the non-normal distribution of the data. Moreover, a univariate analysis (binary logistic regression) was performed to detect factors associated with language recovery; subsequently, a multivariate logistic regression analysis was conducted using variables with p values < 0.05 from the univariate analysis. The results are presented as odds ratios (OR), 95% confidence intervals (95% CI), and p values. A p value < 0.05 was considered statistically significant. A split-half reliability analysis was performed by dividing the cohort into two subsets based on odd versus even patient IDs, and repeating the multivariable analysis in each subset.

## Results

**Fig. 1:**
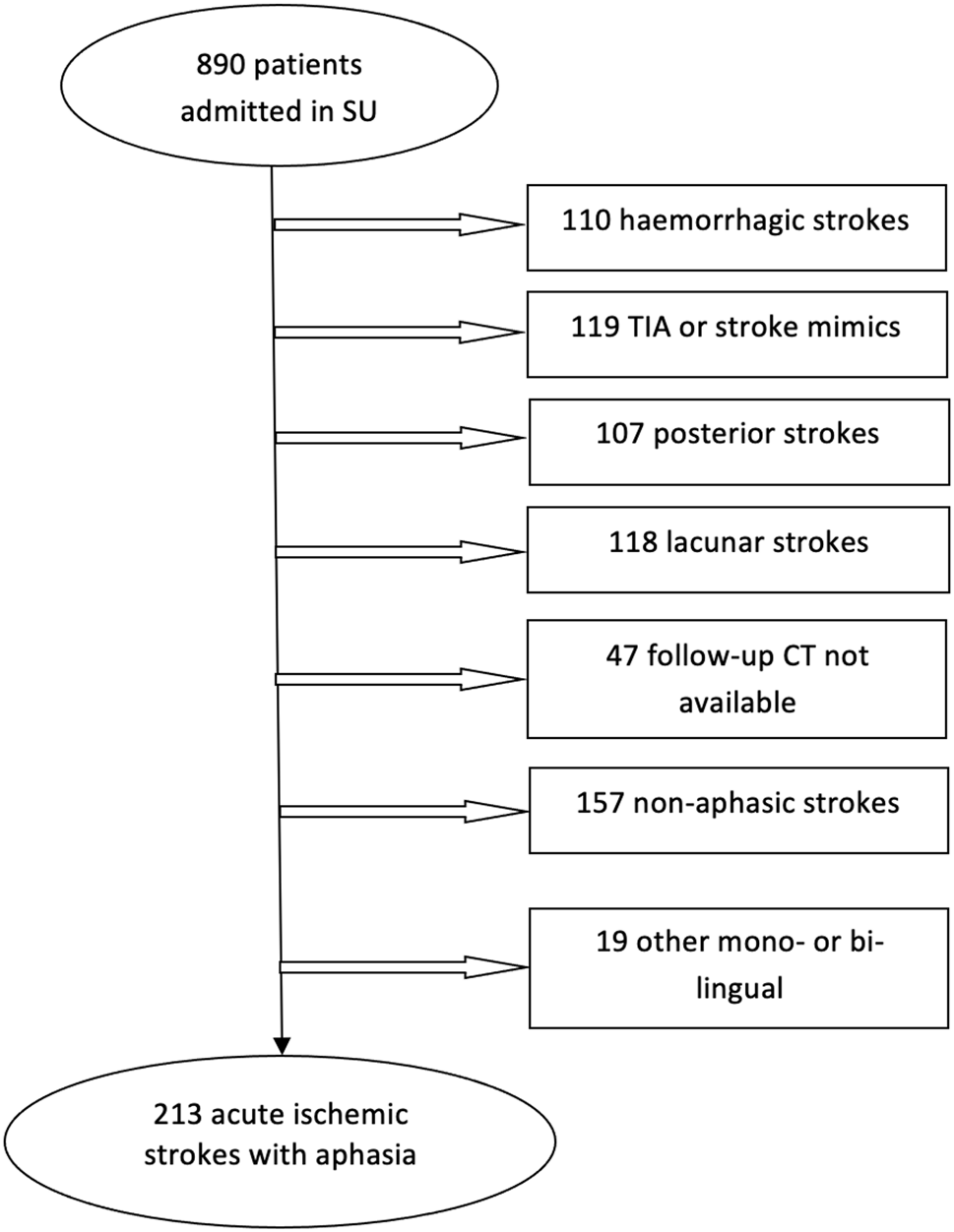
Study flow diagram

From January 2018 to April 2020, 890 patients were admitted to the Stroke Unit, of whom 213 met the inclusion criteria and were included in the analysis. The study flow diagram is presented in Fig. 1.

A total of 153 patients were monolinguals (ML) and 60 were Slovenian–Italian or Croatian–Italian early bilinguals (eBL). The two cohorts were comparable in terms of demographic characteristics (Table 1), including mean age (ML: 77 ± 12 years; eBL: 76 ± 11 years), sex distribution (ML: 54.2% female; eBL: 51.7% female), and years of education (10 ± 4 years in both groups). Cardiovascular risk factors and comorbidities were also similar between groups, with comparable frequencies of atrial fibrillation (ML: 43.1%; eBL: 43.3%), diabetes mellitus (ML: 24.8%; eBL: 26.7%), hypertension (ML: 76.5%; eBL: 76.7%), dyslipidemia (ML: 59.9%; eBL: 60.0%) and active smoking (ML: 16.4%; eBL: 15.0%).

**Table 1:** Comparison of demographics and clinical characteristics of monolingual patients (ML) vs. early bilingual patients (eBL)

	Monolingual ML (n = 153)	Early bilingual eBL (n = 60)	*p values*
*Demographics*			
Age (mean ± dev st)	77 ± 12	76 ± 11	0.313
Female sex (n, %)	83 (54.2%)	31 (51.7%)	0.762
Education yrs (mean ± dev st)	10 ± 4	10 ± 4	0.395
*Vascular risk factors and clinical status (n, %)*			
Pre-stroke cognitive impairment	19 (12.4%)	7 (11.7%)	0.880
Pre-stroke Indipendence	140 (91.5%)	50 (83.3%)	0.078
Hypertension	117 (76.5%)	46 (76.7%)	0.976
Atrial fibrillation	66 (43.1%)	26 (43.3%)	0.686
Diabetes mellitus	38 (24.8%)	16 (26.7%)	0.796
Dyslipidemia	91 (59.9%)	36 (60.0%)	0.986
Current smoker	25 (16.4%)	9 (15.0%)	0.795
*Stroke characteristics*			
NIHSS admission (median, IQR)	10 (4–20)	8 (5–20)	0.785
NIHSS discharge (median, IQR)	2 (0–13)	2 (1–5)	0.662
Ischemic volume mL (median, IQR)	25.0 (5.1–61.5)	32.3 (11.2–76.0)	0.353
*Reperfusive treatment (n, %)*			
Any treatment	103 (67.3%)	37 (62.7%)	0.521
Thrombolysis	57 (37.2%)	22 (36.7%)	0.583
Primary thrombectomy	10 (6.5%)	1 (1.7%)	0.152
Thrombolysis and thrombectomy	36 (23.5%)	14 (23.3%)	0.641
Onset to treatment time (median, IQR)	200 (72–264)	205 (74–270)	0.564
*Haemorraghic trasformation (n, %)*			
Symptomatic cerebral hemorrhage	5 (4.9%)	2 (5.4%)	0.601
*Outcomes*			
Language improvement 7 days/discharge (n, %)	82 (53.6%)	49 (81.7%)	** < 0.001**
mRS at 3 months (median, IQR)	3 (1–5)	2 (1–5)	0.579
Discharge home (n, %)	50 (32.7%)	24 (40.0%)	0.339
90-days mortality	29 (18.9%)	10 (16.7%)	0.844

Data are presented as medians (IQR), and frequencies when appropriated

Pre-Stoke independence defined as mRS ≤ 2. Symptomatic Hemorrhagic transformation defined according to SITS-MOST criteria with % referred to patients who underwent a reperfusive treatment

**Fig. 2:**
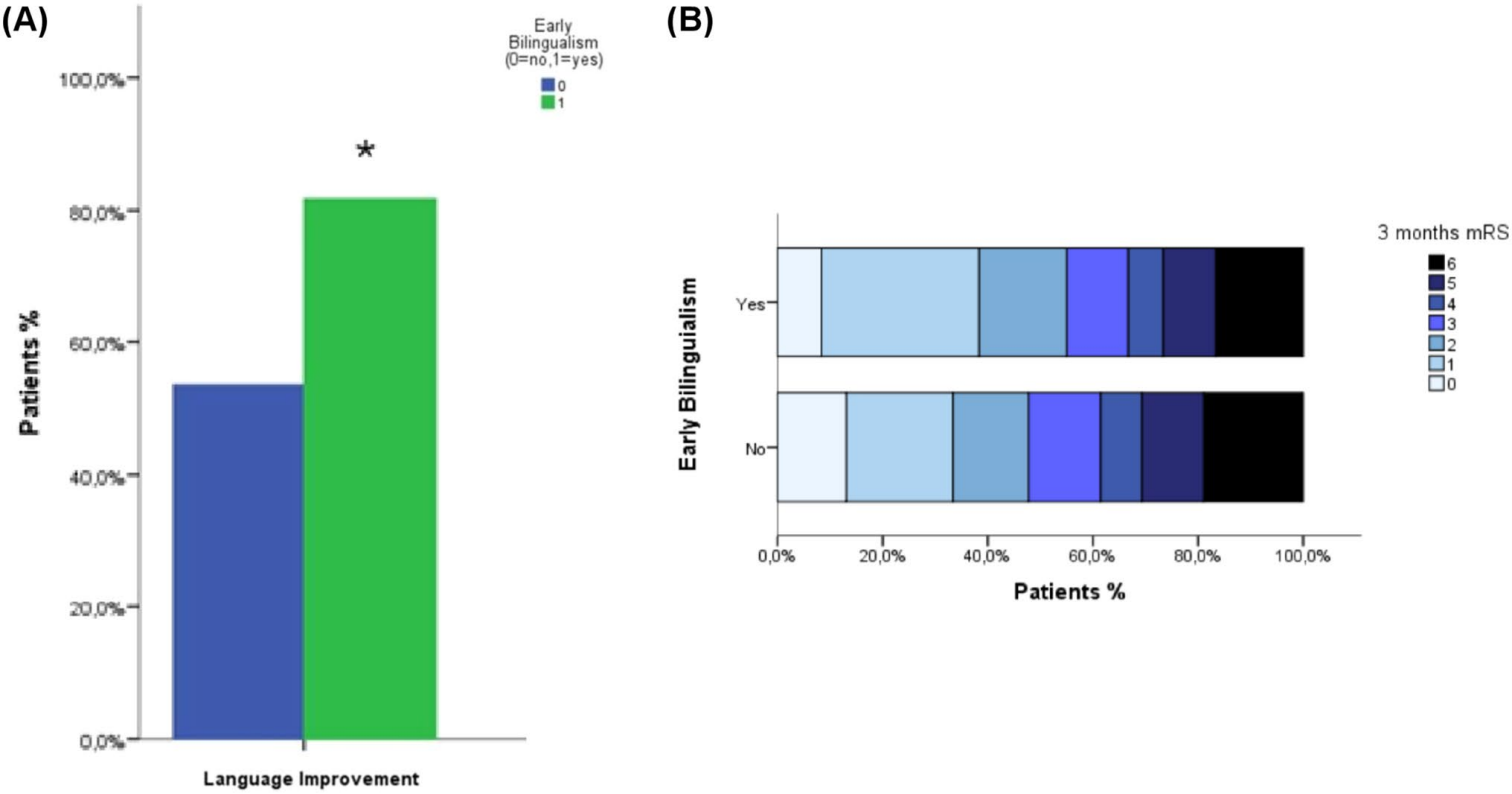
**A** Proportion of patients showing language improvement at discharge (0 = no improvement, 1 = improvement) stratified by early bilingualism (green = early bilinguals; blue = monolinguals). Early bilingual patients exhibited a higher rate of early language improvement. **B** Distribution of 3-month mRS scores according to early bilingualism (yes/no). Each bar represents the percentage of patients reaching a given mRS category

The two groups also showed no statistically significant differences in acute reperfusion therapies or treatment timings (intravenous thrombolysis in 37.3% of ML and 36.7% of eBL patients; mechanical thrombectomy in 6.5% of ML and 1.7% of eBL; and combined therapy to 23.5% in ML and 23.3% of eBL). Median onset-to-treatment time was similar (ML: 200 min; eBL: 205 min). Symptomatic intracerebral hemorrhage according to the SITS-MOST definition [18] occurred in 5 ML patients (4.9%) and 2 eBL patients (5.4%) who received reperfusion therapy. Both groups showed no differences in terms of clinical presentation according to the Oxfordshire Community Stroke Project classification; among ML patients, 39.2% presented with TACS and 60.8% with PACS, compared with 41.7% and 58.3%, respectively, in the eBL group. Median final infarct volume was comparable between cohorts (ML: 25 mL [IQR 5.1–61.5]; eBL: 32.3 mL [IQR 11.2–76.0]). Overall, global neurological severity was comparable between groups at both admission and discharge, with similar NIHSS scores at admission (ML: median 10, IQR 4–20; eBL: median 8, IQR 5–20) and at discharge (ML: median 2, IQR 0–13; eBL: median 2, IQR 1–5). Functional disability was likewise similar (median mRS at 3 months 3 in ML, median mRS at 3 months 2 in eBL, p = 0.579). Notably, early bilingual patients demonstrated a significantly higher rate of aphasia improvement (81.7%) compared with monolinguals (53.6%) (p < 0.001). This association is illustrated in Fig. 2A, which compares the proportion of ML and eBL patients showing early language recovery. Functional outcomes at 90 days in terms of mRS distribution did not differ between groups. Figures 2B illustrate the distribution of 3-month mRS scores stratified by language group (ML vs. eBL). Furthermore, supplemental Table 1 shows the Spearman correlations between key clinical variables.

### Prediction of aphasia recovery

Univariate regression analysis identified several factors potentially associated with aphasia recovery, including age, pre-stroke independence, final ischaemic volume, and motor improvement (p < 0.05). When these variables were entered into the multivariate model, only bilingualism (OR = 4.720, p = 0.005) and motor improvement (OR = 2.510, p = 0.050) remained independent predictors of language recovery (Table 2). The effect sizes observed in the split-half analyses were consistent with those obtained in the full cohort, supporting the internal validity of the findings. Bilingualism remained a stable and significant predictor across all analyses, whereas motor improvement showed a less consistent association, in line with its borderline significance in the full model.

**Table 2:** Logistic multivariate regression for prediction of aphasia recovery at 7 days

Variable	OR	Cl—Lower	Cl—Upper	***p-values***
Bilingualism	4.720	1.590	13.890	**0.005**
Age	1.006	0.971	1.042	0.723
Pre-stroke independence	0.898	0.205	3.940	0.887
Ischemic volume (mL)	0.999	0.995	1.003	0.767
Motor improvement	2.510	1.000	6.290	**0.050**

Multivariate analysis for aphasia recovery. Bold values for *p* < 0.05

## Discussion

Early bilingualism (eBL) is an independent predictor of aphasia recovery within the first seven days after stroke. This result support the view that early bilingualism, defined as the simultaneous acquisition and routine use of both languages from preschool age, contributes to enhanced neural plasticity. Factors such as frequency of language use and phonological or grammatical similarity between languages further shape the neural mechanisms supporting language processing and recovery. Prior research has shown that bilingualism is associated with both structural and functional adaptations of grey and white matter, as well as modifications in language-control and executive-control networks [21, 22]. In particular, linguistic competence and early language acquisition have been associated with increased grey matter density in the left inferior parietal cortex and with greater efficiency of neural networks [23]. These adaptations may reflect either anatomically distinct neural systems for each language, as proposed by the neural divergence theory, or procedurally distinct but interconnected representations within shared brain regions, as suggested by the convergence theory [8, 24]. Language control during bilingual processing engages a distributed network, including the anterior cingulate cortex (for conflict monitoring between competing languages), the dorsolateral prefrontal cortex (for inhibitory control and switching), and the left lateral caudate nucleus (for lexical selection) [25]. Given these neural adaptations, bilingualism has been recognized as a protective factor for cognitive function in ageing [13], as a contributor to delayed dementia onset [12], and as an independent predictor of post-stroke cognitive outcomes [26]. Moreover, converging evidence from neuroimaging and cognitive neuroscience indicates that the age of second-language acquisition plays a fundamental role in shaping the functional organization and efficiency of language and control networks in the bilingual brain [14, 15]. Early bilinguals exhibit stronger interhemispheric functional connectivity, particularly between homologous inferior frontal regions, and greater integration with fronto-parietal executive control networks [14]. In contrast, late bilinguals show reduced interhemispheric connectivity, and display increased unilateral left-hemisphere activation during linguistic tasks, consistent with compensatory recruitment [14]. On this basis, it can be hypothesized that early simultaneous bilingualism promotes more resilient, distributed and efficient language-control networks in the acute post-stroke phase, potentially supported by contralateral compensatory mechanisms.

In our study, early bilingualism emerged as the strongest independent predictor of early language improvement (OR 4.720, p = 0.005), even after adjusting for age, pre-stroke independence, ischaemic lesion volume, and motor improvement. In this context, our findings align with previous evidence showing that, in chronic post-stroke aphasia, bilingual patients exhibit greater language improvement than monolinguals, with outcomes influenced by premorbid proficiency, time to treatment, and the size and location of the focal lesion [9].

Within this framework, beyond the independent contribution of bilingualism to language recovery, our results also identify motor improvement as an independent predictor of early aphasia recovery, even if just reaching significance in the multivariate analysis. This finding supports the hypothesis that post-stroke reorganization of motor networks may improve linguistic reorganization, potentially through the recruitment of shared or tightly interconnected neural circuits linking motor and language systems.

Although motor-language coupling has been investigated in the context of motor neurorehabilitation [27], our understanding of its role in aphasia recovery remains limited. It is noteworthy that the two groups were highly comparable at baseline, as reflected by their demographic characteristics, vascular risk profiles, pre-stroke functional independence, cognitive reserve, and stroke features. Consequently, the observed effect of early bilingualism is reinforced by the clinical homogeneity of the cohort, as differences in language recovery cannot be explained by variability in disease severity or pre-stroke status.

### Strenghts and limitations

This study has some limitations. First, it is a retrospective analysis, which may be subject to the influence of unmeasured confounding factors. Second, data were collected from a single Stroke Unit, which may limit the generalizability of the findings. Third, language performance was assessed using the NIHSS language item administered in Italian for all patients. Consequently, a qualitative analysis of spontaneous speech was not available, and cross-language intrusions or code-switching phenomena could not be systematically evaluated. Finally, another limitation of this study is the lack of a detailed analysis of lesion topography. Imaging data were obtained from follow-up NECT, which allowed estimation of infarct volume; however, a systematic analysis of lesion topography in relation to language-related networks was not performed in the present study.

Despite these limitations, our study demonstrates that early bilingualism independently predicts aphasia recovery within the first seven days after anterior-circulation stroke in a large clinical cohort. These findings support the development of new rehabilitation strategies in the acute phase and raise important questions regarding the neural plasticity mechanisms that may confer a linguistic advantage in bilingual patients. Future research should aim to identify radiological and anatomical markers in bilingual patients with ischemic stroke in order to determine whether the involvement of specific language-related networks influences recovery trajectories. In addition, prospective studies integrating detailed neuroimaging and linguistic assessments are needed to clarify the potential differential effects of bilingualism on post-stroke language recovery.

## Conclusion

Early bilingualism (eBL) is an independent predictor of aphasia recovery within the first seven days after stroke. This finding supports the hypothesis that early bilingualism enhances neural plasticity and facilitates post-stroke language recovery. In the acute phase after stroke, these effects may be mediated by more effective contralesional compensatory mechanisms in the early bilingual brain.

## Supplementary Information

Below is the link to the electronic supplementary material.

Supplementary file1 (DOCX 16 KB)

## Data Availability

The data will only be made available from the corresponding author upon reasonable request.

## Declarations

### Guarantor

Paolo Manganotti.
