# Supplementary material for: Early bilingualism as a protective factor against acute post-stroke aphasia
Source: J Neurol. 2026 Apr 27;273(5):291. doi: 10.1007/s00415-026-13820-2 (PMC13121373; doi:10.1007/s00415-026-13820-2)
Supplement: Supplementary file 1 — Supplementary file1 (DOCX 16 KB) [file 415_2026_13820_MOESM1_ESM.docx]

**Supplemental table 1.** Spearman’s correlation matrix (ρ) among clinical variables

| **Variables** | **Volume (mL)** | **NIHSS discharge** | **NIHSS at admission** | **Age** | **Education (Years)** |
| --- | --- | --- | --- | --- | --- |
| **Volume (mL)** | 1.000 | **0.578*** | **0.530*** | 0.118 | –0.048 |
| **NIHSS discharge** | **0.578*** | 1.000 | **0.651*** | **0.206*** | –0.143 |
| **NIHSS admission** | **0.530*** | **0.651*** | 1.000 | **0.190*** | –0.145 |
| **Age** | 0.118 | **0.206*** | **0.190*** | 1.000 | **–0.279*** |
| **Education** | –0.048 | –0.143 | –0.145 | **–0.279*** | 1.000 |

**Notes:** * p value<0.05
